# Endoscopic Vitrectomy Combined with 3D Heads-Up Viewing System in Treating Traumatic Ocular Injury

**DOI:** 10.1155/2024/9294165

**Published:** 2024-07-08

**Authors:** Yuan-Shao Cheng, Chung-Hao Hsiao, Wei-Ping Hsia, Hung-Ju Chen, Chia-Jen Chang

**Affiliations:** ^1^ Department of Ophthalmology Taichung Veterans General Hospital, Taichung, Taiwan; ^2^ Department of Optometry Central Taiwan University of Science and Technology, Taichung, Taiwan

## Abstract

**Purpose:**

To investigate effects and complications of endoscopic vitrectomy combined with 3D heads-up viewing system in treating traumatic ocular injury. *Patients and Methods*. This is a retrospective interventional case series in a tertiary referral center in Taiwan, and we included patients of traumatic ocular injury, and they underwent endoscopic vitrectomy combined with a 3D heads-up viewing system.

**Results:**

Fourteen eyes of traumatic globe injury from 14 patients were studied over a 30-month period. Preoperative VA ranged from no light perception (NLP) to 6/6. Postoperative visual acuity improved in 11 of the 14 eyes (79%). Until 6 months after surgery, all eyes had attached retina. The median logMAR BCVA was 2.4 at the first visit and 1.19 at the last visit (*p* = 0.0028). No subject suffered from retinal detachment, endophthalmitis, or other severe complications.

**Conclusions:**

Vitrectomy using endoscopy combined with 3D heads-up viewing system allowed early evaluation and intervention in traumatic ocular injuries. Most of our cases showed both anatomical and visual acuity improvements.

## 1. Introduction

Traumatic ocular injury is a common cause of ocular morbidity and permanent visual impairment worldwide [1–4]. It requires urgent treatment because blindness not only affects life quality but also productivity [5]. For open globe injuries involving the posterior segment, the sooner surgical intervention is performed, the better the prognosis [6]. In the acute stage, vision is often highly compromised due to the concurrent presence of corneal edema, inflammatory membranes, or hemorrhage. For eyes with an opaque cornea following ocular trauma, endoscopic surgery allows an earlier diagnosis and prevents retinal pathology such as retinal damage and detachment.

Three-dimensional (3D) surgery system consists of a high dynamic range camera installed on a microscope with a device for 3D display. The surgeon who operates it wears a pair of polarized glasses. Two images are merged and then separated horizontally through the polarized glasses so that each eye sees a slightly disparate image, producing depth perception with the two eyes. A 3D surgery system allows the surgeon to perform the procedure in a more comfortable “Heads‐Up” position than when looking through the microscope [7, 8]. It is used in both anterior and posterior ophthalmic surgical procedures. It is considered a safe and ergonomic operation system for ophthalmic surgery [9].

Currently, the 3D heads-up viewing system for vitreoretinal surgery is becoming increasingly popular. However, reports on the combined endoscope and 3D viewing system for vitrectomy are limited. Here, we aim to investigate the effects of vitrectomy with endoscopy combined with the 3D heads-up viewing system in the treatment of traumatic ocular injuries.

## 2. Patients and Methods

This is a single-site, retrospective case series. We collected clinical data of patients visiting a tertiary referral center in Taiwan, the Taichung Veterans General Hospital, during the period from November 1, 2019, through May 1, 2021. This study adhered to the tenets of the Declaration of Helsinki. All patients were clearly informed about the use of endoscope and the 3D surgical system for the operation and related risks. All patients signed the informed consent form on the operation. All patients' information was anonymized prior to analysis.

The inclusion criteria for this study were patients with traumatic ocular injuries who underwent endoscopic vitrectomy with a 3D head-up system from November 1, 2019 to May 1, 2021. The exclusion criteria were patients with critical condition requiring life support, clinical follow-up period less than 6 months, preexisting retinal diseases, and age under 18 years old.

The recorded data included patient demographics, trauma history, operative findings, surgery procedures, and ophthalmic examination from the initial to the final follow-up. The eye examination included assessment of visual acuity using Snellen visual acuity chart, relative afferent pupillary defect (RAPD), and anterior segment and posterior segment examination. Eyes were categorized based on ocular trauma score (OTS) as follows: category 1 (0–44), category 2 (45–65), category 3 (66–80), category 4 (81–91), and category 5 (92–100) [10].

Surgical procedures were performed using 23-gauge or 25-gauge three-port pars plana vitrectomy (CONSTELLATION Vision System, Alcon Laboratories, Inc.) under a microscope with a noncontact wide-field visualization system. Specifically, a NGENUITY 3D visualization system (Alcon Laboratories, Inc.) was attached to a microscope (Carl Zeiss Meditec) for observing all surgical procedures. During the surgery, the surgeon wore a pair of passive 3D polarized glasses and positioned himself about 2 meters away from the screen. The endoscope system was Endo Optiks E4 (Endo Optiks, Inc., Little Silver, NJ, USA).

**Figure 1:**
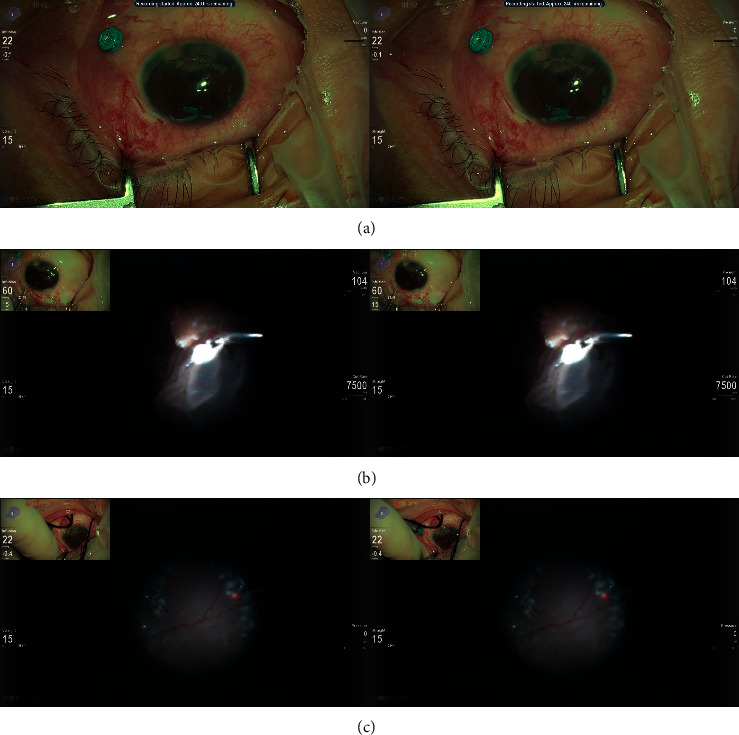
The intraoperative image from patient no. 5 (a 45-year-old man suffering from chronic trauma with corneal scar and pupil synechia). Two images on the monitor were merged horizontally into polarized 3D glasses to create 3-dimensional image (a). Both the endoscopic image and the wide-angle view image were integrated on one monitor screen, which make the surgeon know the direction more clearly when performing endoscopic surgery. Retinal detachment with PVR changes was noted by endoscopic image (b). The retina was reattached and laser photocoagulation was applied. 3D endoscope visualization system offers a good vision in an air-filled eye (c).

The vitrectomy technique entailed a 23-gauge/25-gauge three-port pars plana approach involving a sclerotomy located 3 to 3.5 mm posterior to the limbus. If the patient had traumatic cataract or lens dislocation, we performed lensectomy with complete removal of the capsular bag but no lens implantation. Core vitrectomy was performed and the peripheral vitreous was carefully shaved using scleral depression. In the case of intraocular foreign body (IOFB), the foreign body was removed by forceps. In the patient of IOL dislocation, suture IOL was performed. The air-fluid exchange was done in the event of retinal detachment, and the subretinal fluid was drained out. The subretinal proliferations were extracted from the retinotomy if it was difficult to flatten the retina after air-fluid exchange. Endolaser photocoagulation was applied whenever needed. Silicone oil or gas tamponade was performed at the end of the surgery if necessary. In the patient of proliferative vitreoretinopathy (PVR), encircling scleral buckle was placed. The representative image is shown in Figure 1.

Statistical analyses were conducted using the Statistical Package for the Social Sciences (IBM SPSS version 22.0; International Business Machines Corp, New York, USA). Snellen or best-corrected visual acuity (BCVA) values were converted into the logarithm of the minimum angle of resolution (logMAR) for statistical analysis. The following logMAR assignments were used for BCVA worse than 1/200: counting fingers (CF) = 2.0, hand motion (HM) = 2.3, light perception (LP) = 2.7, and no light perception (NLP) = 3. Preoperative and postoperative visual acuities were compared using the Wilcoxon signed-rank test. The difference in final visual acuity between the OTS group and our series was analyzed using the chi-square test. Preoperative visual acuity worse than 1/200 rates was compared with postoperative visual acuity worse than 1/200 rates using the McNemar test. A *p* value of <0.05 was considered statistically significant.

## 3. Results

Fourteen eyes from 14 subjects over a 30-month period were studied (Table 1). The majority of the patients were male (78.6%), and the mean age was 46.2 ± 18.3 years (range, 25–79 years). Comorbidities included intraocular foreign body, retinal detachment, choroidal detachment, IOL dislocation, lens dislocation, and proliferative vitreoretinopathy. Preoperative VA ranged from no light perception (NLP) to 6/6.

During the first surgery, tamponade was performed using silicone oil on 6 eyes (43%) and SF6 gas on 2 eyes (14%). Lensectomy was performed on 6 eyes. Another combined procedure included iridoplasty (2 eyes), suture IOL (1 eye), encircling scleral buckle (1 eye), and IOFB removal (3 eyes).

### 3.1. Anatomical Success

Until 6 months after surgery, all eyes (100%) showed attached retina. Most retinae were reattached without tamponade. During the initial surgery, 6 eyes were filled with silicone oil and 2 eyes were filled with SF6 gas. Silicone oil was removed in 3 eyes and the remaining 3 eyes was filled with silicone oil until the last follow-up visit.

### 3.2. Visual Acuity and OTS

The median logMAR BCVA was 2.4 at the first visit and 1.19 at the last visit (*p* = 0.0028, by the Wilcoxon signed-rank test). A total of 11 cases (79%) had visual acuity less than 1/200 on presentation. After calculating the ocular trauma score (OTS), patients were categorized in group 1 of 8 eyes and group 2 of 6 eyes. Improvements in postoperative visual acuity were found with 11 of the 14 eyes (79%) (Table 2). Two eyes still showed good visual acuity (6/6) after 6 months. There was one eye initially suffered from open globe injury, retinal detachment, and submacular hemorrhage. The retina was reattached after treatment, but the vision remains poor with light perception due to optic atrophy. Final VA distributions based on OTS categories are presented in Table 3. There is a significant difference in final visual acuity between the OTS group and our series, as shown in Table 4.

### 3.3. Complications

We found no intraoperative complication, nor postoperative complication like retinal detachment, endophthalmitis, and other severe complications.

## 4. Discussion

The ocular trauma score (OTS) is a system widely used to predict the visual prognosis of patients with traumatic ocular injury. All of the injured eyes included in this study belonged to the more serious and poorer prognosis group (OTS categories 1 and 2). In OTS, only 3% of category 1 patients was predicted to achieve visual acuity 6/60 or greater. However, in our study, 30% of patients achieved visual acuity 6/60. Similarly, 50% of category 2 patients in our study had visual acuity greater than 6/60, compared with 28% predicted by OTS. The difference was statistically significant, as indicated in Table 4. The reason we think why the visual prognosis of patients in our study is better than that predicted by OTS is that early intervention can improve the prognosis of patients with severe ocular trauma. The combination of endoscopy and 3D imaging is a powerful tool to provide us with early interventional surgery.

Vitrectomy is a challenging procedure for surgeons on severely injured eyes with corneal opacity. Temporary keratoprosthesis (TKP) is a useful tool for visualizing the retina during vitrectomy. However, implantation of TKP requires more procedures and that prolongs the operation time. Besides, the open-sky stage increases the risk of suprachoroidal hemorrhage. The intraoperative manipulation and the use of silicone oil are associated with corneal graft failure [11, 12]. Unlike the conventional viewing system, endoscopy can bypass opaque cornea to give a view of the posterior segment, allowing early intervention and preventing late complications after severe ocular trauma.

The endoscopic approach offers significant advantages for vitreoretinal surgery for traumatic cases. Because endoscopes can access the retina and intraocular structures closely, they can provide proximal views of structures of interest, including the ciliary body, pars plana, and peripheral retina [13]. Besides, the endoscopy obviates aggressive scleral indentation, which should be preferably avoided for eyes with recent open globe injuries [14]. Although vitrectomy under air is often effective in terms of retinal stabilization, visualizing the retina is markedly compromised in certain situations [15]. Therefore, the endoscope allows for observation in close proximity to the retina, even under air. Sabti and coworkers reported serial cases of endoscopic-assisted vitrectomy for treating severe ocular trauma. They found that among their 50 eyes with open globe injuries, 36 (83.7%) showed improved vision after surgery [16]. In our present study, we observed anatomical success in all 14 eyes. Additionally, the number of eyes exhibiting visual acuity worse than 1/200 significantly decreased (from 10 eyes to 3 eyes) (McNemar test, *p* = 0.016).

IOFBs increase the risk vision-threatening complications like endophthalmitis, retinal detachment, and toxic optic neuropathy or retinopathy [17]. Immediate removal of IOFBs is important to reduce the risk of endophthalmitis and permanent vision loss [18, 19]. Endoscopy facilitates IOFB removal by direct visualization of difficult-access structures such as the ciliary body, ciliary sulcus, and peripheral retina. It also provides better surgical field to conduct complete vitrectomy, which could reduce pathogenic microorganisms. In the present study, IOFBs were successfully removed in 3 cases. We noted no endophthalmitis with good VA improvements after surgery.

Proliferative vitreoretinopathy (PVR) is an independent risk factor of unfavorable outcome for ocular trauma [20]. Anterior PVR might cause ciliary body fibrosis that subsequently leads to hypotony and phthisis [21]. The endoscope is an ideal tool for assessing ciliary body status and determining whether or not to perform silicone oil filling [22]. Endoscopy is considered important in treating PVR-related retinal detachment concurrent with severe corneal opacity. Kita and coworkers reported a case series of advanced PVR using the endoscopic approach. They found that at the mean follow-up of 8.2 months after first endoscopic vitrectomy, 3 of 4 subjects (75%) had achieved successful retinal reattachment under silicone oil [23]. In our present study, we had one case of chronic trauma with pupil synechia, retinal detachment, and PVR changes (patient no. 5). After operation, his retina was attached under silicone oil. His visual acuity also improved from NLP to HM, despite limited improvement due to macular degeneration.

Incomplete removal of residual vitreous could result in complications like vitreous incarceration and retinal detachment [24]. The 3D heads-up visualization system provides a number of surgery-enhancing advantages such as better depth perception and the use of digital color filters. Such features enhance visualization and improve vitreous removal during surgery [25].

There are some limitations of the endoscopic vitrectomy system, like the lack of stereopsis and the inability to perform bimanual surgery. That is because the surgeon is viewing a screen rather than through two microscope eyepieces. Therefore, there is no stereopsis possible. Non-stereoscopic visual cues are needed for distance perception through shadow and changes in object size (degree of magnification) [26]. Furthermore, the intraocular instruments are difficult to orient under the endoscopic view. The operator should look back to microscope to confirm doubtful positions. The way we had combined the endoscopy with 3D heads-up viewing system allowed integrating both the endoscopic image and the wide-angle view image on one monitor screen. The surgeon could thereby operate without the need to exchange the view from microscopy to the 3D monitor. This helped the surgeon operate more comfortably, efficiently, and safely.

We also conducted a thorough literature search on reports that combined endoscopy with 3D heads-up viewing system. However, we found no relevant literature on this topic. Our present study is the first of its kind in evaluating the performance of vitrectomy using endoscopy combined with a 3D heads-up viewing system in treating traumatic ocular injury. In the acute stage of open globe injuries, an early vitrectomy has risks of fluid leakage from the wound, intraoperative bleeding, and poor visualization of the vitreous cavity. Our results demonstrated that small gauge vitrectomy assisted by endoscopy combined with a 3D heads-up viewing system was less invasive and well visualized, minimizing risks of early intervention.

Our present study has some weaknesses like its retrospective nature, no control group, and variability of eye conditions following trauma and surgical steps. Another limitation of the study is its small sample size. Larger clinical studies are needed to confirm effects of this new approach.

## 5. Conclusion

In modern microincision vitreoretinal surgery, the endoscope and 3D heads-up viewing system are both valuable tools. We here have described a case series of ocular trauma treated with vitrectomy based on endoscopy combined with a 3D heads-up viewing system. Their anatomic outcomes were good. This system allowed early evaluation and intervention in traumatic ocular injury even with an opaque cornea. It provided a precise and complete vitreous clearance during vitrectomy, reducing the risk of anterior proliferation that could result in severe PVR, hypotony, and retinal redetachment.

## Figures and Tables

**Table 1:** Summary of patient characteristics and surgical procedures.

Patient no.	Sex	Age, years	Cause for operation	Anterior segment	Comorbidity	Lens status	Surgery procedure	Combined procedure
1	M	35	Trauma (working)	Central corneal laceration	Cataract, VH	Natural lens	VT + lensectomy	Nil
2	M	25	Trauma (working)	Central corneal laceration	Cataract, VH	Natural lens	VT + lensectomy	Nil
3	M	79	Trauma (falling down)	Iridodialysis	Hyphema, RD (submacular blood), IOL disappeared	s/*p* ECCE	VT + SO	Iridoplasty
4	F	75	Trauma (falling down)	Iridodialysis	Hyphema, RD (macula involved), IOL dislocation	s/*p* phaco	VT + SO	Suture IOL
5	M	46	Chronic trauma	Pupil synechia	RD with PVR	Natural lens	VT + SO	SB (encircling), iridoplasty
6	M	48	Trauma (working)	Central corneal laceration	IOFB, cataract	Natural lens	VT + lensectomy	IOFB removal
7	M	26	Trauma (working)	Central corneal laceration	Lens dislocation, RD (macula involved)	Natural lens	VT + lensectomy + SF6	Nil
8	M	26	Trauma (working)	Central corneal laceration	VH	Natural lens	VT	Nil
9	M	37	Trauma (working)	Clear cornea	IOFB	Natural lens	VT	IOFB removal
10	M	57	Trauma (working)	Clear cornea	IOFB	Natural lens	VT	IOFB removal
11	F	35	Trauma (working)	Central corneal laceration	VH, RD	Natural lens	VT + SO	Nil
12	M	38	Trauma (working)	Central corneal laceration	Hyphema, cataract, RD (submacular blood)	Natural lens	VT + lensectomy + SF6	Nil
13	F	48	Trauma (working)	Central corneal laceration	CD, RD (macula involved), cataract	Natural lens	VT + lensectomy + SO	Nil
14	M	72	Trauma (falling down)	Central corneal laceration	Hyphema, RD (submacular blood)	s/*p* phaco	VT + SO	Nil

M = male; F = female; PVR = proliferative vitreoretinopathy; VH = vitreous hemorrhage; RD = retinal detachment; CD = choroidal detachment; IOL = intraocular lens; SO = silicone oil tamponade; SF6 = sulfur hexafluoride (SF6) tamponade; IOFB = intraocular foreign body; ECCE = extracapsular cataract extraction; phaco = phacoemulsification; VT = vitrectomy; SB = scleral buckle; CF = counting fingers; LP = light perception; NLP = no light perception.

**Table 2:** Summary of patient's VA, OTS, and 6- month retina status.

Patient no.	Preoperative VA	OTS	OTS category	Postoperative VA					6 months retina
				1D	2W	1M	3M	6M	
1	LP	47	2	LP	CF	CF	CF	CF	Attached
2	HM	47	2	CF	1/60	1/60	3/60	3/60	Attached
3	LP	26	1	LP	LP	LP	LP	HM	Attached, silicone oil tamponade macular degeneration
4	LP	36	1	LP	HM	CF	1/60	1/60	Attached
5	NLP	16	1	NLP	LP	LP	HM	HM	Attached, macular degeneration
6	2/60	43	1	2/60	2/60 (6/30)	2/60 (6/60)	6/15	6/15	Attached
7	HM	36	1	HM	HM	1/60 (6/60)	1/60 (6/60)	1/60 (6/60)	Attached
8	HM	47	2	HM	CF	6/7.5	6/7.5	6/7.5	Attached
9	6/5	63	2	6/7.5	6/6	6/5	6/6	6/6	Attached
10	6/6	63	2	6/6	6/6	6/6	6/6	6/6	Attached
11	HM	36	1	HM	CF	5/60 (NC)	6/60 (NC)	6/60 (NC)	Attached
12	CF	46	2	3/60	5/60	6/60	4/60	5/60	Attached
13	HM	36	1	HM	CF	1/60	2/60	3/60	Attached, silicone oil tamponade
14	LP	36	1	LP	HM	LP	LP	LP	Attached, silicone oil tamponade, optic atrophy

VA = visual acuity; OTS = ocular trauma score; NV = neovascularization; D = day; W = week; M = month; LP = light perception; HM = hand motion; CF = counting fingers; NLP = no light perception.

**Table 3:** Comparison of final visual acuity distributions between OTS prediction and our series.

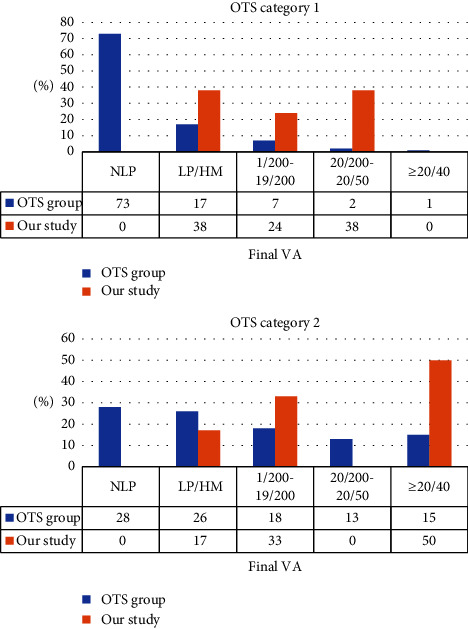

**Table 4:** Comparison of final visual acuity between OTS prediction and our series.

	OTS group	Our study	*p*value^*∗*^
OTS category 1			<0.001
NLP	73 (73.0%)	0 (0.0%)	
LP/HM	17 (17.0%)	38 (38.0%)	
1/200−19/200	7 (7.0%)	24 (24.0%)	
20/200−20/50	2 (2.0%)	38 (38.0%)	
≥20/40	1 (1.0%)	0 (0.0%)	
OTS category 2			<0.001
NLP	28 (28.0%)	0 (0.0%)	
LP/HM	26 (26.0%)	17 (17.0%)	
1/200–19/200	18 (18.0%)	33 (33.0%)	
20/200–20/50	13 (13.0%)	0 (0.0%)	
≥20/40	15 (15.0%)	50 (50.0%)	

^*∗*^Chi-square test.

## Data Availability

The clinical data used to support the findings of this study are available from the corresponding author upon request.
